# Norwegian nursing students’ evaluation of vSim® for Nursing

**DOI:** 10.1186/s41077-018-0070-9

**Published:** 2018-06-19

**Authors:** Ingrid Tjoflåt, Tone Knutsen Brandeggen, Ellen Synnøve Strandberg, Dagrunn Nåden Dyrstad, Sissel Eikeland Husebø

**Affiliations:** 1 Department of Surgery, University Hospital, Stavanger, Norway; 2 Sissel Eikeland Husebø, Stavanger, Norway

**Keywords:** Virtual simulation, vSim® for Nursing, Nursing, Education

## Abstract

**Background:**

vSim® for Nursing is the first web-based platform linked to the nursing education curriculum. It is an American simulation tool, developed in 2014 through a collaboration between Wolters Kluwer Health, Laerdal Medical and the National League for Nursing. To our knowledge, no studies have evaluated vSim® for Nursing from the nursing students’ perspective in Norway. The aim of the study was to evaluate second year Norwegian nursing students’ experiences with the virtual clinical simulation scenario in surgical nursing from vSim® for Nursing.

**Methods:**

A descriptive and a convergent mixed method design was utilised. The method comprised a 7-item questionnaire with five open-ended questions. Sixty-five nursing students participated in the study.

**Results:**

The majority of Norwegian nursing students evaluated the virtual clinical scenario in surgical nursing from vSim® for Nursing useful, realistic and educational in preparing for clinical placement in surgical care. However, a small portion of the nursing students had trouble understanding and navigating the American vSim® for Nursing program.

**Conclusions:**

Introducing virtual simulation tools into the nursing education encompasses faculty and student preparation, guidance from faculty members during the simulation session and support for students who are facing difficulties with the simulation program.

## Background

The bachelor of nursing programme in Norway offers theoretical and practical education. It is regulated by the national curriculum and is delivered in Norwegian [1]. Half of the programme is comprised of studies in clinical placements, including surgical, medical and psychiatric wards, as well as in nursing homes and municipal health care services. Throughout the program, various digital learning and advanced simulation tools are used to optimise learning [2]. The use of simulation has been shown to be beneficial and effective for nursing students in their learning processes [3–5]. Indeed, advances in technology are creating new innovative approaches, including simulation and web-based learning, which offer new pedagogic possibilities related to flexibility, interactivity and motivation for learning. The students of today expect digital, interactive and flexible solutions that help to prepare them for real-world patient care experiences [6]. Virtual clinical simulation is an emerging technology that has been suggested to be effective in nursing education [7–10]. Virtual reality simulation is defined as ‘The use of computer technology to create an interactive three dimensional world in which the objects have a sense of spatial presence; virtual environment and virtual world are synonyms for virtual reality’ [11].

A variety of virtual simulation products have been developed [12]. One of these virtual simulation tools is vSim® for Nursing, an American simulation tool, which was developed in 2014 through a collaboration between Wolters Kluwer Health, Lippincott, Laerdal Medical and the National League for Nursing. vSim® for Nursing is the first web-based simulation platform linked to the nursing education curriculum. It was established to simulate clinical nursing scenarios and includes fully integrated learning resources [13]. Nursing students have the opportunity to interact with virtual patients and receive direct feedback on their performance and actions. The nursing scenarios can help the students to apply knowledge, make decisions, complete interventions, get feedback on their actions and repeat activities and content at their own pace [14]. vSim® for Nursing is organised in terms of learning objectives, planning, complexity and cues [9, 10, 15, 16]. Virtual clinical simulation for nursing students is used in various ways: as innovative classroom pedagogy to teach certain concepts and as a supplemental teaching strategy related to knowledge and skill acquisition and for preparation prior entering clinical practice settings [7–10, 14]. Although research on vSim® for Nursing is limited, preliminary evidence demonstrates that it enhances nursing students’ knowledge, and it has been positively reviewed by students and faculty members [9, 16]. To our knowledge, no studies have evaluated vSim® for Nursing from the nursing students’ perspective in Norway. Therefore, the aim of this study was to evaluate second year nursing students’ experiences with a virtual clinical simulation scenario in surgical nursing from vSim® for Nursing.

## Methods

The study follows a descriptive and convergent mixed method design QUAN/QUAL, in which quantitative and qualitative data are collected simultaneously, with equal priority [17, 18]. The quantitative data were collected using a 7-item questionnaire with response options on a 5-point Likert scale (1 representing strongly agree and 5 representing completely disagree). Additionally, the questionnaire included five open-ended questions. The results from the open-ended questions were used to validate and enhance the results from the closed-ended questions. [17].

### The questionnaire

The design of the questionnaire was inspired based on previous research focusing on students’ satisfaction with vSim® for Nursing [9]. The first author developed the first draft of the questionnaire. Further, it was discussed and revised several times by the university faculty team involved in the vSim® for Nursing simulation session and evaluation, before it was finalised. Table 1.provides a detailed description of the questionnaire.

**Table 1:** Questionnaire—evaluation of vSim® for Nursing

Age: ……				
Male: ……	Female: ……..			
A. How will you evaluate your computer skills? Please circle or cross around the answer you want to enter.				
Very good	Good	Medium	Satisfactory	Mediocre
B. How will you evaluate your English knowledge? Please circle or cross around the answer you want to enter.				
Very good	Good	Medium	Satisfactory	Mediocre
Please indicate to what extent the following statements are in accordance with your opinion. The rating categories are: Strongly agree, Slightly agree, Neither agree or disagree, Slightly disagree, Completely disagree. Please circle or cross around the answer you want to enter.				
1. vSim® was easy to navigate.				
Strongly agree	Slightly agree	Neither agree or disagree	Slightly disagree	Completely disagree
2. It was motivating to work with vSim®.				
Strongly agree	Slightly agree	Neither agree or disagree	Slightly disagree	Completely disagree
vSim® was useful to learn new knowledge in surgical nursing care.				
Strongly agree	Slightly agree	Neither agree or disagree	Slightly disagree	Completely disagree
vSim® was useful for reinforcing knowledge in surgical nursing care.				
Strongly agree	Slightly agree	Neither agree or disagree	Slightly disagree	Completely disagree
To work with vSim® was a good preparation for clinical practice.				
Strongly agree	Slightly agree	Neither agree or disagree	Slightly disagree	Completely disagree
The content of vSim® was relevant for my role as a nurse.				
Strongly agree	Slightly agree	Neither agree or disagree	Slightly disagree	Completely disagree
3. The vSim® provided me with different learning possibilities that promoted learning in surgical nursing care.				
Strongly agree	Slightly agree	Neither agree or disagree	Slightly disagree	Completely disagree

The last part of the questionnaire included the five open-ended questions. The questions asked the students to describe their experience participating in the vSim® simulation, to elaborate on their positive and potentially negative experiences and to indicate whether they recommended the virtual simulation for future use and an explanation of the reason why. Moreover, one question asked the students to share additional comments regarding their virtual simulation experiences.

### Ethics and participants

The study was approved by the Norwegian Centre for Research Data (NSD no: 54963), and permission was given by a university located in Norway. The study was carried out in a classroom in October 2017. Written and oral information about the study was provided to the students. Confidentiality and the voluntary nature of the research were emphasised as well as the fact that the students’ answers would have no impact on them or their study programme.

Sixty-five nursing students in the second year bachelor programme for nursing were invited to participate in the evaluation of the virtual simulation scenario. All participants signed a letter of consent. All 65 students completed the evaluation forms, resulting in a response rate of 100%. The consent form was collected separately from the questionnaire. To maintain the confidentiality; each questionnaire was provided with a number. Moreover, the coded questionnaires were stored separately from the consent forms.

### The virtual simulation and evaluation session

The two hourly virtual simulation session and the evaluation took place during a mandatory preparation week before the clinical placement studies in surgical wards. Thus far, virtual scenarios had not been introduced to the nursing students in the current educational curriculum. Introducing and evaluating vSim® for Nursing in the preparation week provided an opportunity to gain valuable knowledge about Norwegian nursing students’ experiences.

To avoid spending time on practical issues during the 2-h simulation session, a licence and instructions concerning the practical use of vSim® for Nursing were provided in an e-mail to the students 1 day in advance. In addition, one of the faculty members provided brief plenary information about the session in advance of the simulation session. During the simulation session, the students were instructed to follow the vSim® format and complete the pre-test on post-operative care before entering the virtual simulation scenario. The pre-test is a component of the vSim® for Nursing program. Since vSim® for Nursing is an American tool delivered in English, a list of medications used in the scenario was provided to the students to facilitate the simulation.

As this was the students’ first experience with vSim® for Nursing, they worked in pairs to allow for discussions and interactive learning. A team including faculty members from the university and a clinical tutor from the hospital guided the students in the vSim® simulation and evaluation session.

One surgical scenario from vSim® for Nursing was chosen for the students to complete during the surgical simulation. The patient in the simulation scenario was a young female who had undergone surgery for a ruptured appendix and required post-operative care. This particular nursing scenario was selected since it was the most relevant surgical case related to the Norwegian health care system. The students carried out the simulation, reviewing real-time feedback on what they did. Many of the students performed the simulation scenario twice within the allocated time frame. At the end of the session, the students also completed the post-test which is a component of the vSim® programme.

During the last 20 min of the two hourly sessions, the participants were asked to complete the questionnaire evaluating their experiences working with vSim®. The students placed the completed questionnaire on a table, and the faculty collected these afterward.

### Data analysis

The quantitative data were analysed using the SPSS25 statistical package and included descriptive statistics. Two missing scores distributed among two participants were replaced by the mean value of the relevant item and included in the statistics. Otherwise, all questionnaires were completed. Qualitative content analyses were performed on the open-ended questions [19, 20]. First, the faculty group read the qualitative data independently to obtain an overall understanding, and the meaning units that emerged from the text were identified. Further, the identified meaning units were discussed among all the authors until a consensus was reached. The meaning units were condensed to preserve the core meaning and then further organised into categories and subcategories. Common patterns were compared to identify the students’ experiences with the vSim® for Nursing virtual clinical simulation scenario. The analysis identified two categories related to the nursing students’ experiences. These categories describe the qualitative data on a manifest level and with a low degree of interpretation [19, 20]. The qualitative data in the study validated and enhanced the understanding of the students’ experiences with vSim® for Nursing [17].

## Results

In this section, nursing students’ characteristics are first presented and then the results from the QUAN and QUAL parts of the questionnaire are presented independently.

### Nursing students characteristics

Sixty-five second year nursing students with a mean age of 24 years (19–49 years) responded to the questionnaire. The majority of the nursing students were female (91 %). Almost all (98%) of the students evaluated their computer knowledge either to be very good (15%), good (54%), medium (21%) or satisfactory (8%), while only 2 % rated their computer knowledge to be mediocre. The majority of the nursing students reported that their English knowledge was very good (16%), good (34%), medium (31%) or satisfactory (11%), while only 8% evaluated their English knowledge to be mediocre.

### QUAN data

The majority of the nursing students (40% strongly agree, 23% agree) reported that working with vSim® for Nursing was motivating, promoted learning (31% strongly agree, 35% agree) and was useful for gaining new knowledge (39% strongly agree, 34% agree) as well as for reinforcing knowledge about surgical nursing care (51% strongly agree, 29% agree) (Table 2). The students stated that working with the virtual simulation was a good preparation for their clinical placement studies in surgical wards (46% strongly agree, 25% agree) and that the content of the virtual simulation was directly relevant to their role as a nurse (48% strongly agree, 35% agree). Although some students (*n* = 21) reported difficulties in navigating in vSim®, the majority of them (14% strongly agree, 43% agree) reported that the product was easy to use. The majority of the students (79%) recommended the virtual simulation for future use.

**Table 2:** Nursing students’ (*n* = 65) evaluations of vSim® for Nursing in frequencies and percentages

Evaluation items	Strongly agree *n* (%)	Slightly agree *n* (%)	Neither agree or disagree *n* (%)	Slightly disagree *n* (%)	Completely disagree *n* (%)
Easy to navigate	9 (14)	28 (43)	7 (11)	18 (28)	3 (4)
Motivating	26 (40)	15 (23)	10 (15.5)	10 (15.5)	4 (6)
Learn new knowledge	25 (38)	22 (34)	9 (14)	5 (8)	4 (6)
Reinforce knowledge	33 (51)	19 (29)	5 (8)	5 (8)	2 (4)
Preparation for clinical practice	30 (46)	16 (25)	9 (14)	6 (9)	4 (6)
Relevant for role as nurse	31 (48)	23 (35)	3 (4)	6 (9)	2 (4)
Promoted learning	20 (31)	23 (35)	10 (15)	7 (11)	5 (8)

The results also showed that students found that the virtual simulation was not so easy to navigate and did not contribute, reinforce or promote learning. Almost one third of the students (28% slightly disagree, 4% completely disagree) stated that the product was challenging to use. Working with vSim® was reported to be demotivating (15.5% slightly disagree, 6% completely disagree), neither a useful tool for learning new knowledge (8% slightly disagree, 6% completely disagree), neither reinforcing knowledge learnt previously (5% slightly disagree, 4% completely disagree) and neither promoting learning (11% slightly disagree, 8% completely agree). The results also revealed some students did not find the vSim® product relevant for their role as a nurse (9% slightly disagree, 4% completely disagree). Twenty percent of the students did not recommend the virtual simulation vSim® for Nursing for future use.

### QUAL data

The analysis of the qualitative data related to the nursing students’ experiences with the virtual clinical simulation scenario identified the following two categories: ‘Realistic and useful’ and ‘Difficult and minimal learning’.

### Realistic and useful

The qualitative data revealed that working with the virtual simulation scenario was funny, realistic and highly instructive, aiding the students in learning new knowledge and reinforcing and preparing them for the clinical placement studies in surgical wards.

One student wrote, ‘It is realistic to see a patient and you can choose observations and procedures that are relevant… better than just talking about a case’ (P50).

Another student also expressed a positive experience: ‘Funny and creative way to get to know surgical nursing care; very good learning’ (P14).

The students reported that the virtual simulation was comprised of several tasks that provided them with different possibilities to learn about surgical nursing care. For instance, one stated, ‘Varied, many possibilities related to the product and funny way to find the right solutions on your own’ (P 47). Another student said the simulation was ‘Easy to navigate and a very instructive way to evaluate how we performed the nursing care to the patient’ (P57).

Two students reported that working together in pairs with vSim® was instructive: ‘It was funny and motivating. It is also useful to work together in pairs; the discussion promoted learning’ (P49).

### Difficult and minimal learning

The students who found vSim® difficult and experienced minimal learning did not recommend the virtual simulation for future use. They stated that vSim® was difficult to understand due to the English language being used. One student wrote that it was demotivating and a waste of time, and several others reported that it was difficult to navigate in vSim®. For instance, one student reported it was ‘Difficult with all the information about the patient; we have to go back and forward to find out what we should give to the patient and what to do’ (P14). Another student expressed the following: ‘The system was very slow, a waste of time; we have to go to the ward to learn’ (P41).

Additionally, the difficulties were related to the fact that vSim® for Nursing is provided in English, and some students described that it would be better if the virtual simulation were presented in Norwegian. One student said, ‘The English language made it very challenging, a bit messy set up’ (P 40). Another student stated, ‘I did not understand the whole point. Difficult English and difficult to understand what we should do’ (P58).

A few students described that they did not understand the concept of vSim® and expressed that the product would have been good if they had understood what they were supposed to do. One student said, ‘I thought it was very unclear what the point of the simulation was’ (P25).

## Discussion

The aim of the study was to evaluate second year nursing students’ experiences with a virtual clinical simulation scenario in surgical nursing from vSim® for Nursing.

The results demonstrate that the majority of the students experienced the simulation to be realistic and useful with regard to both learning new knowledge and reinforcing prior knowledge as well as a good preparation for their clinical placement studies in the surgical wards. Most of the students also recommended vSim® for Nursing for future use. These results are in accordance with previous findings from virtual simulation studies in nursing education [9, 16]. The results may reflect that the participants in this study were young nursing students who expected new, innovative and varied digital learning possibilities that help them to prepare for real-world patient care experiences [6]. The fact that the simulation scenario used in the session was selected by the faculty team may explain why the students found it motivating and relevant as a preparation for clinical placement in surgical care.

On the one hand, the students worked together in pairs during the virtual simulation sessions, which allowed for discussion and promoted learning [21]. On the other hand, the results demonstrated that some of the students participating in the study did not find the virtual simulation to be educational. Those students experienced difficulties using the tool, and they reported minimal learning. Even though the majority of the nursing students evaluated their English language to be very good, good and satisfactory, one of the unexpected difficulties that was reported was that vSim® for Nursing was in English. These results may indicate that it is not only the students’ knowledge in English that is important for gaining or consolidating knowledge when working with a virtual surgical nursing care scenario. Another explanation for the students’ difficulties might be the fact that it was the first time that the students had worked with a virtual simulation scenario, and the time allocated for the session was too short to gain familiarity with the tool. Moreover, the faculty team that provided guidance during the session may have overlooked the students’ problems. Although working in pairs promoted learning, student groups having trouble with vSim® for Nursing may likely influence each other negatively resulting in minimal learning.

### Study limitations

Several limitations must be addressed. The study sample was small and involved only one bachelor of nursing programme in Norway. Hence, the findings cannot be generalised but offer additional and international perspectives about the acceptability and feasibility of the program. The faculty guided the students during the vSim® for Nursing simulation session and collected the questionnaire from the nursing students during the 2-h simulation session. This may have influenced the students to answer in a way that they thought was expected, which could question the trustworthiness of the study [22]. However, the questionnaire was anonymous, and it was clearly communicated to the students that participating in the study would have no effect on their study progress. Another factor that could influence the credibility of the results is that the questionnaire was not tested for reliability and validity but constructed based on previous research on vSim® for Nursing [9, 16]. Finally, the study did not use a pre- and post-test to assess the effectiveness of vSim® for Nursing, nor did the study reveal if the students would transfer the knowledge gained from the vSim® for Nursing simulation to clinical post-operative practice in surgical wards. However, a formal evaluation of the students’ knowledge might have affected the students’ experience of the vSim® for Nursing.

## Conclusion

Norwegian nursing students’ evaluations of a virtual clinical simulation in surgical nursing demonstrate that most of them found the vSim® for Nursing to be useful and educational in preparing for clinical placement in surgical care. However, a small portion of the nursing students emphasised that the English language as well as navigating in the programme presented difficulties. The study revealed that introducing virtual simulation tools in nursing education to optimise preparation for clinical placement should include adequate time for faculty and student preparation. Additionally, guidance from faculty members is critical during the simulation session as well as support for students who are encountering difficulties with the simulation programme. The nuances of language, medication names as well as clinical practices differ across countries so localisation of such products is recommended to maximise impact for transferring learning to clinical practice.
